# Removing the Polyanionic Cargo Requirement for Assembly of Alphavirus Core-Like Particles to Make an Empty Alphavirus Core

**DOI:** 10.3390/v12080846

**Published:** 2020-08-03

**Authors:** Julie M. Button, Suchetana Mukhopadhyay

**Affiliations:** 1 Department of Molecular and Cellular Biochemistry, Indiana University, Bloomington, IN 47405, USA; jmbutton@iu.edu; 2 Department of Biology, Indiana University, Bloomington, IN 47405, USA

**Keywords:** self-assembly, virus capsid, non-nucleic acid cargo

## Abstract

The assembly of alphavirus nucleocapsid cores requires electrostatic interactions between the positively charged N-terminus of the capsid protein (CP) and the encapsidated polyanionic cargo. This system differs from many other viruses that can self-assemble particles in the absence of cargo, or form “empty” particles. We hypothesized that the introduction of a mutant, anionic CP could replace the need for charged cargo during assembly. In this work, we produced a CP mutant, Minus 38 (M38), where all N-terminal charged residues are negatively-charged. When wild-type (WT) and M38 CPs were mixed, they assembled into core-like particles (CLPs). These “empty” particles were of similar size and morphology to WT CLPs assembled with DNA cargo, but did not contain nucleic acid. When DNA cargo was added to the assembly mixture, the amount of M38 CP that was assembled into CLPs decreased, but was not fully excluded from the CLPs, suggesting that M38 competes with DNA to interact with WT CPs. The composition of CLPs can be tuned by altering the order of addition of M38 CP, WT CP, and DNA cargo. The ability to produce alphavirus CLPs that contain a range of amounts of encapsidated cargo, including none, introduces a new platform for packaging cargo for delivery or imaging purposes.

## 1. Introduction 

Alphaviruses are enveloped, positive-sense RNA viruses that are members of the Togaviridae family [1]. The virion has three concentric layers: (1) a nucleocapsid core consisting of the RNA genome surrounded by 240 copies of the capsid protein (CP) to form the inner-most shell, (2) a host-derived lipid membrane surrounding that core, and (3) 80 trimeric glycoprotein spikes, comprised of the E2 and E1 proteins, that are embedded in the lipid bilayer and present on the surface of the complete virion [2,3]. In many mature particles, the glycoprotein spikes and core have aligned icosahedral symmetries, a result of the interactions between the internal and external layers of the particle [4,5,6,7,8]. The CP has two domains with unique functions: a disordered, positively-charged N-terminal domain that interacts with the negatively-charged genome to form cores during early assembly [9,10,11,12], and an ordered C-terminal domain that interacts with neighboring C-terminal domains and with the E2 protein during the later budding stage [13,14,15,16]. The N-terminus has also been predicted to contain a coiled-coil motif [17,18]. Empty nucleocapsid cores, or those not containing any genome, have not been observed, suggesting that genomic material is necessary for successful alphavirus core assembly. One hypothetical model of core assembly is that the viral RNA acts to neutralize the cationic N-terminus of the CP and provide a scaffold to hold CP monomers together until a full core is assembled [19].

An in vitro self-assembly system can be used to study the early stages of alphavirus nucleocapsid assembly that occur prior to interactions with the E2 protein. Using this system, studies have shown how the CP-nucleic acid and CP-CP interactions influence core assembly [9,19,20,21,22,23,24,25,26,27]. Alphavirus CPs only self-assemble into core-like particles (CLPs) in vitro in the presence of polyanionic cargo [20,26,28]. These CLPs recapitulate both the structure and function of nucleocapsid cores that are formed during a viral infection [20,25,29,30]. CLPs have been assembled around single-stranded DNA (ssDNA), RNA, gold nanoparticles, and negatively-charged small molecules [20,26,31]. However, double-stranded DNA (dsDNA) or RNA cannot initiate CLP assembly [20]. Thus, it is apparent that the cargo used during CLP assembly can vary, suggesting no particular structure or sequence is necessary, and that assembly is initially driven by electrostatic interactions between CPs and the cargo. In vitro, the charge-neutralizing and scaffold roles of the cargo can be modulated by adjusting cargo length. A shorter cargo, such as a 27-mer ssDNA oligo primarily neutralizes the CP while a longer cargo, such as a 48-mer ssDNA oligo neutralizes and scaffolds CP monomers [19]. 

Although alphaviruses require polyanionic cargo for assembly, many viral systems do not. These viral systems can be induced to form empty cores in vitro by modulating solution conditions including pH [32,33], ionic strength [32,33], and temperature [34,35,36]. In some systems, the capsid or core protein can be truncated or modified to allow for assembly in the absence of nucleic acid. For example, cowpea chlorotic mottle virus forms different sized particles by fusing an elastin-like peptide to the N-terminus of the CP and increasing the temperature during assembly [37]. Alternatively, Hepatitis B virus CP that lacks the RNA-binding domain allows for the assembly of capsids in buffers with high ionic strength and/or temperature [38]. In Rous sarcoma virus, replacing the Gag nucleocapsid domain with the GCN4 dimerization domain enables the assembly of virus particles that do not require nucleic acid [39]. These examples of protein engineering demonstrate how adding to, removing, or exchanging part of the capsid protein can result in a significant change in assembly. 

It was recently shown that a Ross River virus (RRV) CP, in which four key lysine residues are replaced by aspartate residues (4D CP), can self-assemble CLPs in the absence of cargo, albeit at very low yields. The 4D cargo-free CLPs are smaller than CLPs formed with wild-type (WT) CP + DNA cargo and are less sensitive to disassembly by high ionic strength and temperatures relative to WT + DNA cargo CLPs, suggesting that they have a different structure [19]. Based on these initial findings, we sought to design a CLP system where mutated CPs could replace the need for cargo, enabling construction of cargo-free CLPs, that could potentially serve as a drug delivery platform. We hypothesized that a mutant CP with a net-negative N-terminus might eliminate the need for nucleic acid cargo to initiate assembly. We mutated all of the positively-charged residues in the WT CP N-terminus to negatively-charged residues to make a mutant CP called Minus 38 (M38). When WT CP and M38 CP were mixed, they assembled into CLPs in the absence of nucleic acid cargo. These CLPs were of similar size and morphology to WT + DNA cargo CLPs. When nucleic acid was added to the WT + M38 assembly system, we determined that M38 competes with nucleic acid for CLP assembly. In addition, the quantity of M38 incorporated into the CLPs could be modulated by the order of addition of the components. 

## 2. Methods

### 2.1. Cloning the Minus 38 Capsid Plasmid

**Figure 1:**
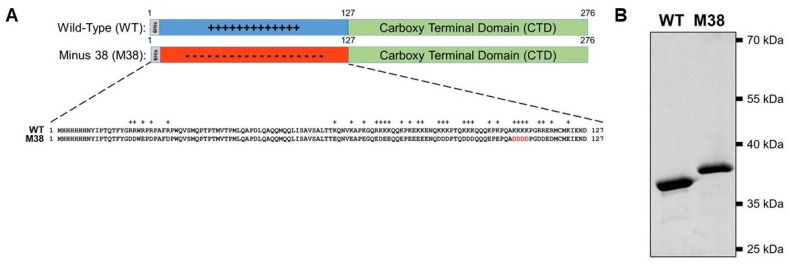
Ross River Virus (RRV) wild-type (WT) and Minus 38 (M38) mutant capsid proteins used to study cargo-free self-assembly. (**A**) Schematic representation of RRV WT capsid protein and M38 capsid protein. Below the schematics is the sequence of the N-terminus of each protein. The four residues in red are the four aspartates that were mutated from lysine in the 4D capsid protein. (**B**) Both WT and M38 capsid proteins can be purified in vitro. Due to their vastly different isoelectric points, the two proteins migrate differently on an SDS-PAGE gel. Three micrograms of each protein was loaded on the gel and the signal detected using 2,2-trichloroethanol (TCE).

The Ross River virus (RRV) mutant capsid protein (CP) sequence (AA18-123) was ordered from GeneArt in a pMA-T vector (ThermoFisher Scientific, GeneArt, Hampton, NH, USA). The sequence was amplified using the following primers: (F) 5′-CAACCCAGACTTTTTACGGAGACGATTGGGAGCCTGACCCGGCGTTC-3′ and (R) 5′-GAATATGCAGTCATTCTCGATCTCCATGCACATATCTTCATCATCCCCTGGATC-3′. The amplified sequence was used to replace the sequence for AA18-123 of the pET29b vector (Novagen, Madison, WI, USA) containing the wild-type (WT) RRV CP sequence with a 6-His tag on the N-terminus (pET29b_6HisWTRRVCP) using the QuikChange Lightning Site-Directed Mutagenesis kit (Agilent, Santa Clara, CA, USA), resulting in the amino acid sequences shown in Figure 1. Successful cloning was confirmed by DNA sequencing of the plasmid encoding the CP.

### 2.2. Ross River Virus (RRV) Capsid Protein Expression and Purification

The pET29b_6HisWTRRVCP plasmid was expressed in Rosetta 2 cells (Novagen, Madison, WI, USA). Cells were grown in Luria broth with 34 µg/mL chloramphenicol and 50 µg/mL kanamycin at 37 °C with shaking until an OD_600_ of 0.4–0.6 was reached, at which point isopropyl-β-D-1-thiogalactopyranoside (IPTG) was added to the culture to a final concentration of 1 mM. Cells were grown for an additional 16–20 h at 16 °C with shaking and were then pelleted by centrifugation at ~3100× *g* and 4 °C. The supernatant was discarded, and the resulting pellet was stored at −20 °C until purification. Cells were resuspended on ice in 20 mM sodium phosphate buffer pH 7.4 at a cell density of 0.06–0.08 g/mL with protease inhibitors (2 µg/mL Leupeptin, 2 µg/mL Aprotonin, and 1 mM PMSF), and lysed by 4-to-6 30-s rounds of sonication on ice, with the final 1–2 rounds occurring in 1 M NaCl to disrupt protein-nucleic acid interactions. The cell lysate was clarified by centrifugation at 13,000 rpm and 4 °C for 45 min in a JA-17 rotor. The supernatant was brought up to 60 mM imidazole using buffer B (see below) and then loaded on a HisTrap HP column (GE Healthcare, Princeton, NJ, USA) preconditioned with buffer A (20 mM sodium phosphate buffer, 10 mM imidazole, 1 M NaCl, pH 7.4). The protein was eluted in buffer B (20 mM sodium phosphate buffer, 400 mM imidazole, 1 M NaCl, pH 7.4). The peak fractions were combined and diluted 1:4 by volume in 20 mM sodium phosphate buffer pH 7.4 before being loaded on a HiTrap SP FF column (GE Healthcare, Princeton, NJ, USA) that was preconditioned with buffer C (20 mM HEPES, 250 mM NaCl, 5 mM EDTA, pH 7.4). The protein was eluted in one peak in buffer D (20 mM HEPES, 1.3 M NaCl, 5 mM EDTA, pH 7.4). The peak fractions were concentrated and buffer exchanged into HN buffer (20 mM HEPES, 150mM NaCl, pH 7.4) using a Vivaspin 6 10 kDa MWCO filter (GE Healthcare, Princeton, NJ, USA) at 5000× *g* and 4 °C to 1 mL. The protein concentration was determined using the absorbance at 280 nm using an assumed Abs (1%) of 12.58 g/L. 

The expression and purification of pET29b_6HisMinus38CP was similar to that of pET29b_6HisWTRRVCP, except the protein did not undergo a second round of purification over the SP column. The protein concentration was determined using the absorbance at 280 nm using an Abs (1%) of 12.77 g/L. 

### 2.3. Core-Like Particle (CLP) Assembly Assay

**Figure 2:**
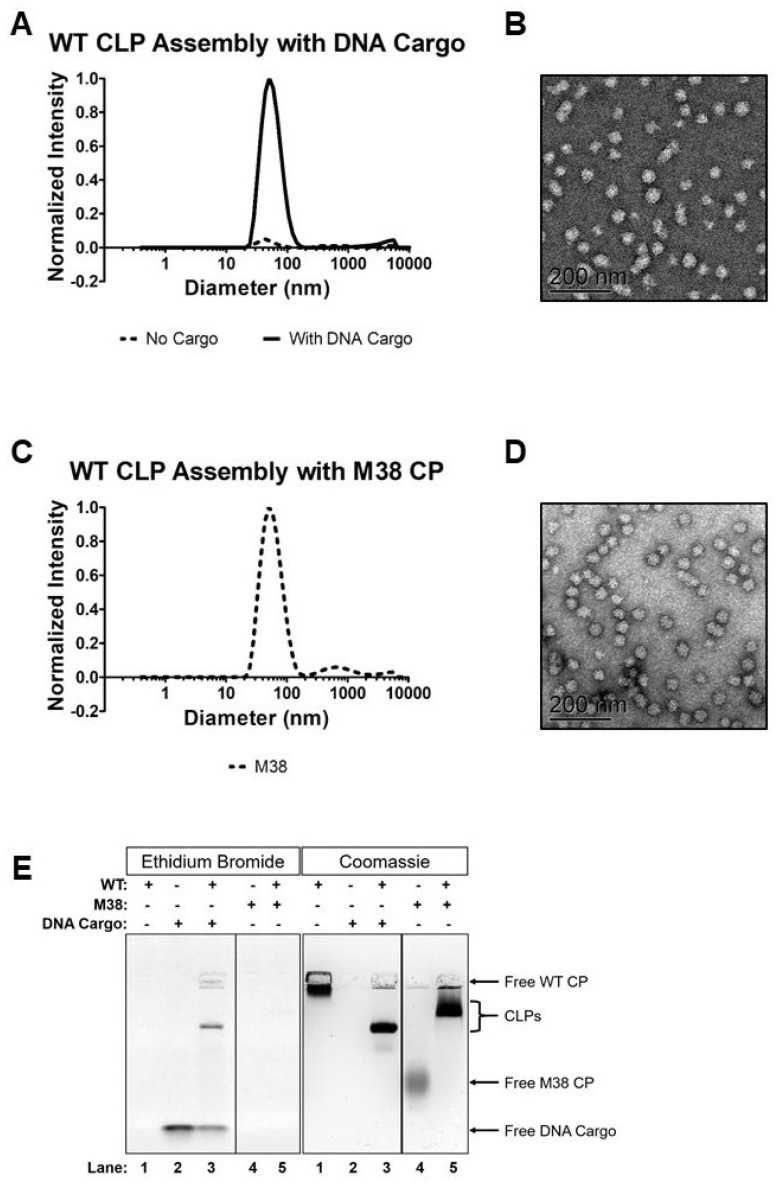
M38 capsid protein (CP) can replace DNA cargo during core-like particles (CLP) assembly with WT CP. (**A**) Assembly of WT + DNA cargo CLPs by dynamic light scattering (DLS) and (**B**) negative-stain transmission electron microscopy (TEM). (**C**) Assembly of WT + M38 CLPs by DLS and (**D**) negative-stain TEM. (**E**) Gel-shift analysis of the reactions in (**A**,**C**) plus the M38 CP- and DNA cargo-only controls. The DLS curves represent the average of at least four biological replicates and were normalized to the largest data point in the data set.

Assembly reactions were prepared by first adding capsid protein (CP) to HE buffer (20 mM HEPES, 0.1 mM EDTA, pH 7.4) and then bringing the NaCl concentration up to the indicated concentrations. In the CLP reactions in Figure 2, the NaCl concentration was 150 mM. Assembly was initiated by adding nucleic acid. Reactions were prepared at 3 µM CP and 3 µM nucleic acid in a volume of 30 µL. The nucleic acid used in these experiments was a 48-mer linear oligomer with the sequence 5′-GTTGACGTCACAGATGAGATATAAAGCATAAGGGACATGTTCAAACGG-3′. In the case of assembly in the absence of cargo, the final concentration of each protein was 3 µM, and M38 CP was added last to initiate assembly. For disassembly reactions, the CLPs were preassembled at low salt, and then the NaCl concentration was brought up to the indicated concentrations. For the competition experiments, simultaneous addition was achieved by mixing M38 CP and 48-mer together prior to adding them to WT CP. For the M38- and cargo-first reactions, the first component was added to WT CP, followed by the second component approximately 5 min later; the reactions were allowed to equilibrate for 2 h before separation by ultracentrifugation. 

### 2.4. Dynamic Light Scattering (DLS)

**Figure 3:**
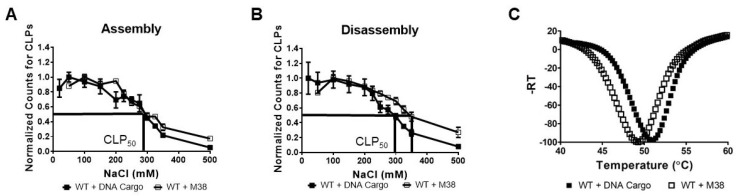
WT + M38 CLPs have similar stability to WT + DNA cargo CLPs. (**A**,**B**) DLS of CLP reactions under (**A**) assembly and (**B**) disassembly conditions. Each curve is an average of three biological replicates with the error bars representing the standard error of the mean (SEM). The method for determining the normalized counts for CLPs is described in the materials and methods. CLP_50_ is the NaCl concentration at which 50% of the maximal CLPs were assembled/disassembled. (**C**) Thermal denaturation of WT + DNA Cargo and WT + M38 CLPs by DSF. Each curve is an average of three biological replicates. WT + DNA Cargo is represented as closed squares and WT + M38 as open squares.

DLS measurements were made on a Malvern Zetasizer Nano ZS instrument (Malvern Panalytical, Malvern, UK) with the attenuator set to 9, the measurement window at 4.2 mm, and the temperature set to 25 °C. A Zen2112 quartz cuvette (Malvern Panalytical, Malvern, UK) was used with a 3-mm path length. Each sample was measured three times; each measurement consisted of 10 runs with each run averaged over 10 s. In Figure 2, the data are graphed with average hydrodynamic diameter (nm) on the x-axis and normalized intensity on the y-axis. Normalized intensity was obtained using the intensity percent for each data point and the derived count rate. In Figure 3, the data are graphed with salt concentration (mM) on the x-axis and the normalized counts on the y-axis. The normalized counts were determined using the intensity percentage for each data point and the derived count rate and then normalized throughout the data points in the curve. The data were analyzed using Microsoft Excel 2013 and GraphPad Prism.

### 2.5. Transmission Electron Microscopy (TEM)

Five microliters of a CLP reaction were placed on a Formvar and carbon-coated 300 mesh grid (Ted Pella Inc., Redding, CA, USA) and stained with 1% uranyl acetate. Stained grids were examined using a JEOL 1010 transmission electron microscope (JEOL USA, Inc., Peabody, MA, USA) operating at 80 kV, and images captured at 25,000× using a Gatan MegaScan 794 charge-coupled-device camera. Images were converted into TIFF format in Digital Micrograph (Gatan Inc., Pleasonton, CA, USA). 

### 2.6. Agarose gel-Shift Assay

CLP assembly reactions were prepared as described in “CLP assembly assay”, then 20 µL of sample was mixed with 4 µL of 6× DNA loading dye (New England Biolabs, Ipswich, MA, USA) and loaded on a 0.8% agarose gel in TBE buffer (45 mM Tris-Base, 0.55% Boric Acid, 1 mM EDTA). The gel was run at 90V (constant) for 45 min and nucleic acids were stained with ethidium bromide. The gel was then soaked in gel drying solution (20% methanol, 10% acetic acid) for at least 10 min, dried on a BioRad Model 583 gel dryer (BioRad, Hercules, CA, USA) at 70 °C for 2 h, and then protein was visualized with Coomassie staining. Images were captured on the BioRad ChemiDoc MP Imaging System (BioRad, Hercules, CA, USA) using the Coomassie setting, and the images were processed in Fiji [40]. 

### 2.7. Differential Scanning Fluorimetry (DSF)

Stability of CLPs was determined by measuring the increase in fluorescence of SYPRO Orange dye (Invitrogen, Waltham, MA, USA). CLPs were prepared as described in “CLP assembly assay”, then 25 µL of sample was combined with 5 µL SYPRO Orange dye at a final concentration of 2.5×. The samples were prepared in MicroAmp Fast Reaction Tubes and capped with MicroAmp Optical 8-cap strips (ThermoFisher Scientific, Hampton, NH, USA). Buffer was used as a negative control. The samples were incubated in StepOnePlus RT PCR System (Applied Biosystems, Foster City, CA, USA) from 25 °C to 99.9 °C continuously in 1 °C increments. The normalized negative first derivative of the fluorescence intensity was graphed as a function of temperature using Microsoft Excel 2013 and GraphPad Prism. 

### 2.8. Purification of Core-Like Particles

Five hundred microliter CLP reactions were prepared at 3 µM CP, 150 mM NaCl, and 3 µM 48-mer linear oligo as indicated. In the mixed capsid CLP reactions, each CP was added at 3 µM. When the molar ratio was varied, only WT CP was changed, and M38 CP and 48-mer DNA remained unchanged at 3 µM. Therefore, for the 0.5:1 and 0.5:1:1 reactions, the WT capsid was at 1.5 µM, whereas for the 2:1 and 2:1:1 reactions, the WT capsid was at 6 µM. Each sample was loaded on a 5–25% linear sucrose gradient in TNE-T buffer (50 mM Tris-HCl pH 7.5, 100 mM NaCl, 1 mM EDTA, 0.1% Triton-X 100), and centrifuged at 32,000 rpm and 4 °C on a SW-41 rotor for 2.5 h. Following centrifugation, 1 mL fractions were collected from the top and CP signal was detected by running the fractions on a 12% SDS-PAGE gel. 

### 2.9. Quantification of Protein by Densitometry

Protein signal on the SDS-PAGE gel was detected using 2,2,2-trichloroethanol (TCE) at 0.5% *v/v* in the resolving gel. The gel was imaged with a BioRad ChemiDoc MP Imaging System I (BioRad Hercules, CA, USA) using the stain-free image setting. Densitometry analysis of protein signal was conducted in Fiji [40].

### 2.10. Quantification of Nucleic Acid by Fluorescence 

For the competition gradients, nucleic acid signal was measured in the fractions using the Quant-iT OliGreen ssDNA Assay Kit (Invitrogen, Hampton, NH, USA) as per the instructions. Briefly, 100 µL of each fraction was combined with 100 µL of 200-fold diluted reagent in a 96-well plate (Greiner Bio-One, Kremsmünster, Austria) and incubated at room temperature for 2–5 min. The nucleic acid stain was excited at 480 nm and emission was measured at 520 nm using a BioTek Synergy H1 plate reader (BioTek, Winooski, VT, USA). A high-range standard curve was run in duplicate on each plate using the oligonucleotide standards provided with the kit. If signal for a sample was outside the standard curve, the sample was diluted in TE buffer (10 mM Tris-HCl, 1 mM EDTA, pH 7.5) until the signal was in the range of the standard curve. 

## 3. Results 

### 3.1. A Negatively-Charged CP Mutant Eliminates the Need for DNA During CLP Assembly

The RRV CP monomer can be expressed and purified as a monomer in a recombinant *E. coli* system (Figure 1A) and, in the presence of polyanionic cargo, subsequently assemble into CLPs [19,20,21,22,23,24,25,26,27]. The N-terminal domain of the RRV CP contains 32 basic amino acids that are largely responsible for interacting with the viral RNA genome (in virions) and with heterologous anionic cargo (in CLPs). In previous work, a stretch of four lysine residues was mutated to four aspartic acid residues (amino acid residues 104–107, Figure 1A) to generate RRV 4D CP. The 4D CP assembled into a small amount of cargo-free particles [19]. Despite the limited extent of assembly, the 4D CP was still able to interact with anionic cargo to form CLPs, demonstrating its ability to assemble both with itself and with nucleic acid cargo. We hypothesized that we could increase the amount of cargo-free CLPs by replacing all of the positively-charged residues in the N-terminus of WT CP with acidic residues, and eliminate the need for DNA cargo during CLP assembly. We mutated the 32 basic residues in the RRV CP N-terminus to aspartic- or glutamic-acid residues, bringing the total number of negatively-charged residues to 38, forming the CP mutant Minus 38 (M38; Figure 1A). The WT and M38 proteins were both expressed and purified from *E. coli* (Figure 1B). M38 migrated slower on an SDS-PAGE gel, presumably because of its low isoelectric point of 4.38 compared to 10.03 for WT CP [41]. 

As expected, the WT CP assembled into CLPs in the presence of a 48-mer ssDNA oligomer, but was unable to assemble in the absence of cargo (Figure 2). Dynamic light scattering (DLS), showed that the WT CP + DNA cargo formed a complex approximately 50 nm in diameter, but CP alone did not form a detectable amount of particles of any size (Figure 2A). Transmission electron microscopy (TEM) confirmed the size (~50 nm) and spherical morphology of the assembled CLPs (Figure 2B). A native agarose gel-shift assay was performed to confirm that the complexes contained both CP and DNA cargo (Figure 2E). In this assay, WT CP (without DNA) did not travel far down the gel and was stained by Coomassie blue, but not ethidium bromide (lane 1, Figure 2E). In contrast, unencapsidated DNA migrated far down the gel and was only stained with ethidium bromide (lane 2, Figure 2E). When CP and DNA cargo were mixed together, the complex migrated to an intermediate position in the gel, and the predominant band was stained with both Coomassie blue and ethidium bromide, reflecting co-migration of both protein and nucleic acid as a complex (lane 3, Figure 2E). 

The addition of M38 CP to WT CP in place of the 48-mer ssDNA oligomer, led to the formation of CLPs of similar size and morphology to those formed from WT CP + DNA cargo (Figure 2). DLS and TEM showed that WT + M38 CLPs had a spherical morphology with a diameter of approximately 50 nm (Figure 2C,D). In the WT + M38 CLPs we also observed a small DLS signal around 500 nm in diameter. These larger particles were not seen by TEM and did not form a prominent band in the gel shift assays, perhaps indicating they are not very stable. It is important to note that there is a bias in DLS for larger sized particles because they scatter more light than the same number of smaller particles. Gel-shift analysis showed that M38 CP migrated into the gel similar to unencapsidated DNA, presumably a result of its overall negative charge. WT + M38 CLPs migrated into the gel, but not as far as WT + DNA (lane 5, Figure 2E, CLPs in bracket). M38 CP, as predicted, did not assemble around negatively charged DNA cargo. Overall, these results suggest that M38 CP, with its highly acidic N-terminus, can replace the need for anionic cargo by assembling directly with WT CP. 

### 3.2. WT + M38 CLPs Have Slight Differences in Stability When Compared to WT + DNA CLPs

Previous work with 4D CP cargo-free CLPs found that these particles were smaller than WT + DNA cargo CLPs. Additionally, 4D CP cargo-free CLPs were more stable in high ionic strength and at higher temperatures compared to WT + DNA cargo CLPs [19]. These results are consistent with structural differences between 4D cargo-free CLPs and WT + DNA cargo CLPs. 

Our CLP assembly studies with WT CP and M38 CP found that the CLPs were similar in size and morphology, suggesting similar structures. We examined the sensitivity of WT + M38 CLPs to ionic strength by studying the effects of ionic strength on CLP assembly (Figure 3A) and disassembly (Figure 3B). CLP assembly in buffer of varying ionic strength was assessed using DLS. We found that the WT + M38 CLPs behaved like WT + DNA CLPs; both had a CLP_50_ of 295 mM NaCl (Figure 3A). The CLP_50_ represents the NaCl concentration at which only half of the maximum CLPs were assembled [27]. In contrast, when monitoring the disassembly of CLPs in response to ionic strength, we found that WT + M38 CLPs had a slightly higher CLP_50_ than WT + DNA cargo CLPs, 345 mM and 295 mM, respectively (Figure 3B). An increase in CLP_50_ between assembly and disassembly is an indicator of capsid hysteresis, a property where capsid assembly and disassembly are not in equilibrium and additional triggers may be required to promote one over the other [42]. Previous work has shown that alphavirus CLPs do not undergo hysteresis during disassembly [19,27], but hysteresis has been observed in the disassembly of capsids in other viral systems [42,43]. The increase in CLP_50_ from 295 mM NaCl during assembly to 345 mM NaCl during disassembly suggests that WT + M38 CLPs may undergo slight hysteresis during disassembly. 

Varying ionic strength during assembly interferes with electrostatic interactions between the N-terminus of WT CP and either DNA cargo or the N-terminus of M38 CP. Another measure of CLP structure and stability is its response to increasing temperature. Using differential scanning fluorimetry (DSF), we found that WT + M38 CLPs showed maximal fluorescence at 49.3 °C, slightly lower than the 50.8 °C for WT +DNA CLPs (Figure 3C). The similarity of the melting temperatures between the two CLPs indicates that there are not significant differences in the hydrophobic interactions in the WT + M38 CLPs compared to the WT + DNA cargo CLPs. 

### 3.3. The Ratio of WT CP and M38 CP Incorporated into CLPs is Determined by input Ratios

**Figure 4:**
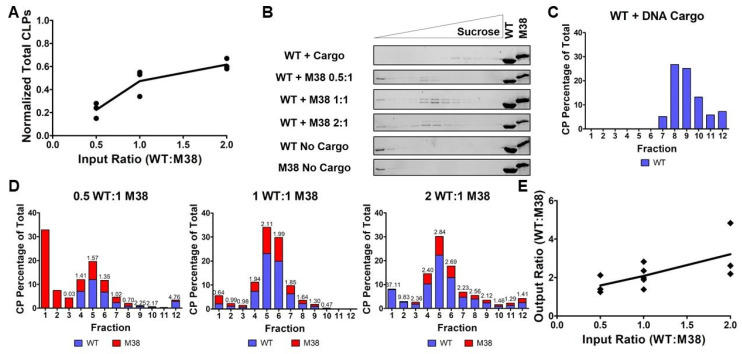
The amount of WT and M38 capsid proteins in the WT + M38 CLPs varies with input ratio. (**A**) As the input ratio of WT:M38 CP increases (by varying WT CP and keeping M38 constant), the number of CLPs formed increases, as determined by DLS. The line represents the mean of the three biological replicates (circles). The numbers are normalized according to Table 1. (**B**) The individual capsid proteins, WT + DNA cargo CLPs, and WT + M38 CLPs were separated over a sucrose gradient using ultracentrifugation. The gel for WT + DNA cargo CLPs is a representative gel of two biological replicates, and the gels for WT + M38 CLPs at all ratios are representative of at least three biological replicates. The control lanes contained 1.5 µg concentrated protein (gels 2 and 4, corresponding to ratios 0.5:1 and 2:1, respectively) or 3 µg concentrated protein (all other gels). (**C**,**D**) Quantification of protein signal from the gels in (**B**) using densitometry (WT CP in blue; M38 CP in red). Each graph represents an average of at least three biological replicates except WT CP + DNA cargo, which represents two replicates; each data point was normalized so that total protein signal in each graph would equal 100%. Above the bars is the ratio of WT:M38 CP in the fraction and is the average of at least three biological replicates. (**E**) As the input ratio increases, the output ratio of the assembled CLPs also increases in a linear fashion. The line represents the mean of the three-to-five biological replicates (diamonds). The output ratios plotted are for fraction 5, as determined in panel (**D**).

For our initial assembly studies, we added equimolar amounts of WT CP and M38 CP to initiate CLP assembly and determined that M38 could replace the need for DNA cargo during CLP assembly. Next, we tested how the molar ratio of added WT CP and M38 CP may affect the characteristics of assembled CLPs. We assembled CLPs by varying the concentration of WT CP but keeping M38 CP constant at 3 µM, as shown in Figure 2 and Figure 3. Using DLS, we found that, as the input ratio (WT:M38) increased from 0.5:1, to 1:1, to 2:1, the total number of CLPs also increased (Table 1 and Figure 4A).

To determine if the different input ratios of WT and M38 CPs affected CLP morphology or composition, samples were separated by centrifugation through a sucrose gradient. When WT + DNA cargo CLPs were applied to the gradient, the CLPs migrated toward the bottom of the gradient, with maximal protein signal at 18.68–20% sucrose, as determined by refractometry (Table 1 and Figure 4C). However, when WT + M38 CLPs were added to the gradient, the CLPs migrated to the center of the gradient, around 14.40–14.63% sucrose (Table 1 and Figure 4B). This difference in sedimentation of WT + DNA CLPs relative to WT + M38 CLPs is likely due to the presence of encapsidated DNA, given that the particle size of WT + DNA and WT + M38 CLPs are similar (Figure 2). Varying the ratio of WT CP and M38 CP in the assembly reaction did not change the sedimentation of the assembled CLPs (Table 1 and Figure 4B). When WT CP or M38 CP were applied individually to the top of a sucrose gradient, in the absence of any cargo, the capsid proteins did not assemble into CLPs or higher-order oligomers, and protein signal remained at the top of the density gradient at approximately 7–12% sucrose (Figure 4B). 

To determine the stoichiometry of the two capsid proteins within the assembled CLPs, we quantified the amount of WT CP in WT + DNA cargo CLPs (Figure 4C) and the ratio of WT CP to M38 CP in each sucrose fraction (Figure 4D). We were able to distinguish WT from M38 CP because these proteins migrate differently on an SDS-PAGE gel (Figure 1B). Using densitometry of the CP bands, we found that, as the input ratio (WT:M38) increased from 0.5:1, to 1:1, to 2:1, the CLP ratio in fraction 5 (the fraction with the most CLPs) increased from 1.57, to 2.11, to 2.84 of WT:M38 CP, respectively (Figure 4C,D). Together, these results suggest that, as more WT CP is added, more CLPs are formed (Figure 4A). Further, the ratio of WT to M38 that is incorporated into CLPs can be modulated by adjusting input ratios (Figure 4B–E and Table 1). 

### 3.4. M38 CP Competes with DNA Cargo to Assemble with WT CP into CLPs 

The prior results suggest that M38 CP behaves like DNA cargo and acts as a nucleating agent to promote assembly with WT CPs into CLPs. We next asked whether CLPs would assemble if all three components—WT CP, M38 CP, and DNA cargo—were added together. We hypothesized that M38 CP and DNA cargo may compete to assemble with WT CP. To test this, we examined the ability of CLPs to form with various input ratios of WT CP to M38 CP in the presence of a 48-mer ssDNA cargo. In these competition experiments, the concentration of WT CP was varied from 1.5 µM, to 3 µM, to 6 µM, and the concentration of M38 CP and DNA cargo were each kept constant at 3 µM. Thus, the ratio of WT CP to M38 CP and DNA cargo ranged from 0.5 to 2. 

**Figure 5:**
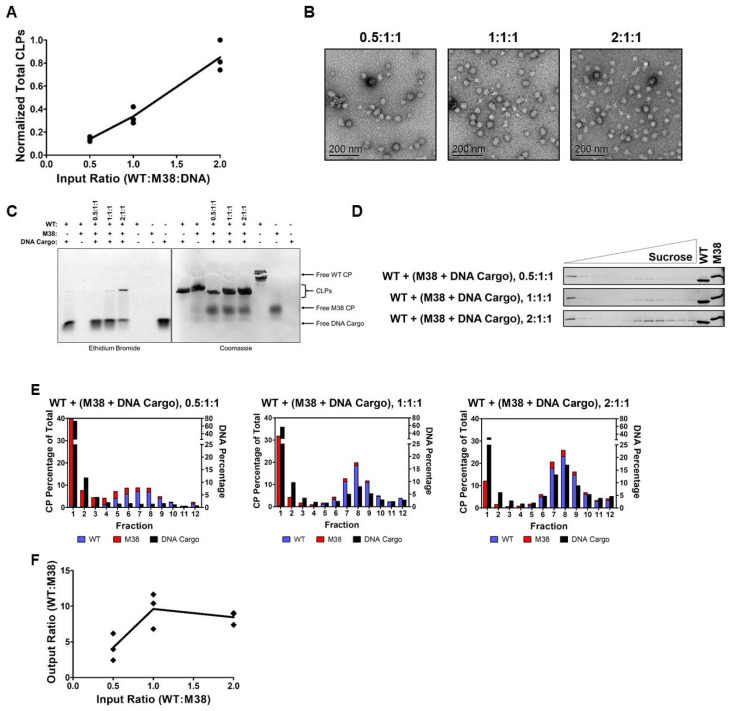
M38 CP competes with DNA for interactions with WT CP and incorporation into CLPs. (**A**) As the input ratio of WT:M38 CP increased, the amount of CLPs formed increased in a linear fashion, as determined by DLS. The line represents the mean of the three biological replicates (circles). The numbers are normalized according to Table 1. (**B**) Negative-stain TEM images of the WT + M38 + DNA cargo CLPs. Addition of DNA cargo did not appear to change the morphology of the CLPs. (**C**) Native gel-shift analysis of the unpurified CLP reactions. The WT + DNA cargo and WT + M38 reactions were assembled at a 1:1 molar ratio and the ratios for the WT + M38 + DNA cargo lanes are shown above the gel. (**D**) Separation of WT + M38 + DNA cargo CLPs over a sucrose gradient by ultracentrifugation. The gels are representative of three biological replicates. The control lanes were loaded with 1.5 µg concentrated protein. (**E**) Quantification of the protein and nucleic acid content in the fractions from the gels in (**D**). Protein content was quantified using densitometry, and nucleic acid content was quantified using fluorescence. Each data point was normalized so that the total protein signal in each graph would equal 100% and total DNA signal would equal 100%. (**F**) As the input ratio of WT:M38:DNA cargo increased, the output ratio increased up to a ratio of 1:1:1, but then plateaued at a ratio of 2:1:1 as determined from the ratio in fraction 8 (peak fraction). The amount of M38 CP incorporated decreased when DNA was added to the CLPs.

When increasing the amount of WT CP in the reaction, the overall quantity of assembled CLPs, as determined by DLS, increased (Table 1 and Figure 5A). TEM confirmed the overall size and morphology of the CLPs is consistent with our other experiments (Figure 5B). Using a gel-shift assay, we observed that CLPs assembled with all three components contained both CP and DNA cargo. However, the gel-shift assay could not differentiate between WT CP and M38 CP in the assembled CLPs (Figure 5C). 

To determine the CP composition of the CLPs, samples were separated on a sucrose gradient, and fractions examined by SDS-PAGE. These experiments produced two interesting observations. First, there was a shift in the CLP peak from 14.40–14.63% sucrose in CLPs without DNA cargo (Figure 4B and Table 1) to 17.62–18.93% sucrose in CLPs with DNA cargo, consistent with an increase in particle mass due to the incorporation of DNA cargo (Figure 5D and Table 1). Quantification of DNA in each fraction confirmed that DNA cargo was present in the shifted fractions (Figure 5E, black bars). Second, both WT CP and M38 CP were detectable in the shifted fractions, demonstrating that both proteins were incorporated into the CLPs. Based on our previous data (Figure 4), if the three-component CLP reaction produced two separate populations of CLPs, (WT + DNA) and (WT + M38), we would have observed two CLP-containing fractions: one at approximately 14% sucrose (without DNA) and the other at approximately 19% sucrose (with DNA) (Figure 5D). 

From the gradient gels and subsequent quantification of the CLP components (Figure 5D,E), we observed that less M38 CP was incorporated in CLPs that also contained DNA cargo relative to WT + M38 CLPs without DNA in the assembly reaction. While M38 CP could compete with DNA for binding to WT CP, it was unable to completely exclude the incorporation of DNA cargo into the CLPs. This was most evident after examining the output ratio of WT CP to M38 CP in the assembled CLPs. In the absence of DNA cargo, the output ratio ranged from approximately 1.5–3 WT CP per M38 CP (Figure 4E). However, in the presence of DNA cargo, this ratio increased to approximately 4-10 WT CP per M38 CP (Figure 5F). This change is primarily due to a reduction of M38 CP incorporated into the CLP. These results suggest that, even though M38 CP can function as a surrogate for nucleic acid cargo in CLP assembly with WT CP, assembly with nucleic acid cargo is competitively favorable. This preference could be due to the number and/or types of interactions occurring between WT CP and the M38 CP versus DNA cargo. 

### 3.5. Order of Addition Affects the Composition but not the Quantity of CLPs Assembled 

We determined that, when M38 CP and DNA cargo were added simultaneously to WT CP, both were incorporated into the resulting CLPs (Figure 5). We next sought to determine how assembly, quantity and composition of CLPs assembled, was affected by order of addition. We tested various orders of addition at constant 1:1:1 molar ratios of WT CP:M38 CP:DNA cargo, each at a final concentration of 3 µM. 

**Figure 6:**
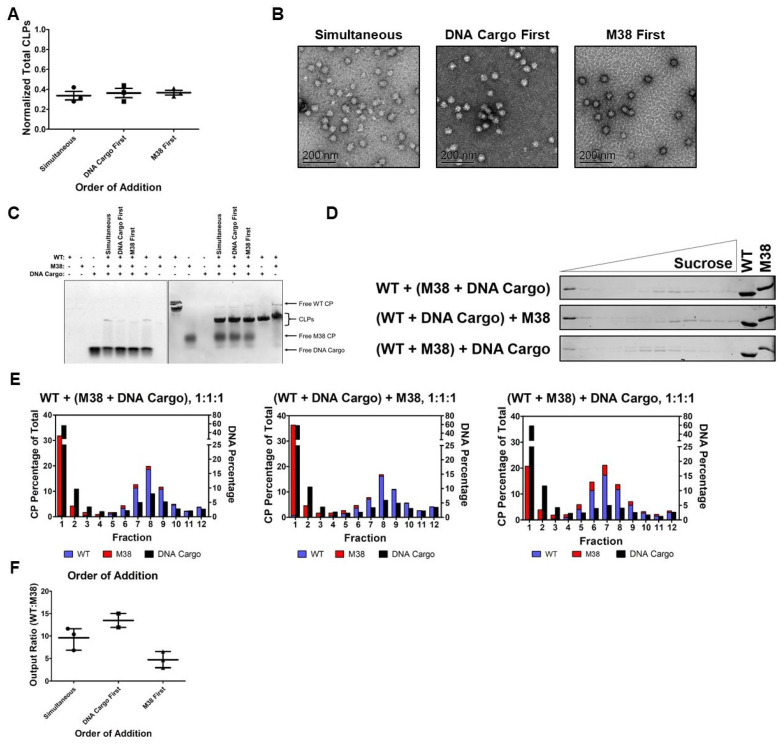
Order of addition affects the composition of the WT + M38 + DNA cargo CLPs. (**A**) The amount of total CLPs formed (determined via DLS) did not change based on the order of addition of M38 CP and DNA cargo at the tested ratio. (**B**) Negative-stain TEM of the WT + M38 + DNA cargo CLPs showing that order of addition of M38 CP and DNA cargo did not alter the morphology of the CLPs. (**C**) Native gel-shift analysis of the unpurified CLP reactions. The WT + DNA cargo and WT + M38 reactions were assembled at a 1:1 molar ratio. The 1 WT + 1 M38 + 1 DNA cargo reactions were assembled in the order indicated above the gel. (**D**) Separation of WT + M38 + DNA cargo CLPs over a sucrose gradient by ultracentrifugation. The gels are representative of three biological replicates. The control lanes are 1.5 µg concentrated protein. (**E**) Quantification of the protein and nucleic acid content in the fractions from the gels in (**D**). Protein content was quantified using densitometry, and nucleic acid content was quantified using fluorescence. Each data point was normalized so that the total protein signal in each graph would equal 100% and the total DNA signal would equal 100%. (**F**) Order of addition of M38 CP and DNA Cargo to WT CP changes the output ratio of WT:M38 CP, with the most M38 CP incorporated when M38 CP was added to WT CP prior to DNA cargo. Ratios are based on fraction 8 for DNA cargo first and simultaneous conditions, and fraction 7 for M38 first. Simultaneous = WT + (M38 + DNA cargo); DNA cargo first = (WT + DNA cargo) + M38; M38 first = (WT + M38) + DNA cargo.

We compared addition of all three components at the same time (simultaneous), the addition of WT CP + DNA cargo followed by M38 CP (DNA cargo first), or the addition of WT CP + M38 CP followed by DNA cargo (M38 first). We found that the total number of resulting CLPs was not affected by the order of addition (Figure 6A and Table 1). Furthermore, the order of addition did not appear to affect the size or morphology of the CLPs (Figure 6B). The gel-shift analyses of these reactions showed that, for all conditions, there was co-migration of CP and nucleic acid, indicating encapsidated DNA in every case. However, we could not determine how much WT CP and M38 CP were incorporated from this result (Figure 6C). 

The CP and DNA cargo composition of these CLPs was analyzed using sucrose gradients and quantification as described above. In the samples where DNA was added first or simultaneously with M38 CP, there was less detectable M38 CP than when it was the first component added to the assembly reaction (Figure 6D). Additionally, there was a shift in the peak fraction for CLPs based on the order of addition. When DNA was added first or simultaneously with M38 CP, the peak CLP fraction was at 18.93–18.94% sucrose, similar to WT + DNA cargo at 18.68–20.00% sucrose (Figure 6D,E, Table 1). However, when M38 CP was added to WT CP prior to DNA (M38 first), the peak CLP fraction shifted up to 17.27% sucrose, indicating less sedimentation, most likely due to incorporation of less DNA cargo (Figure 6D,E, Table 1). When the DNA was added prior to M38 CP (DNA cargo first) or simultaneously with M38 CP, the ratio of WT CP to M38 CP was approximately 9.5–13.5. However, when M38 CP was added to WT CP prior to DNA cargo (M38 first) the ratio decreased to approximately 5 (Figure 6F). Overall, these results indicated that the order of addition of M38 CP and DNA cargo affects the final composition of the resulting CLPs. DNA cargo appears to be preferred over M38 CP, but is not fully able to displace M38 CP if it is added to the reaction mixture last. 

## 4. Discussion

In this work, we used Ross River capsid proteins and found that the highly negatively charged M38 CP can act as a substitute for DNA cargo to promote CLP assembly with WT CP. The resulting WT + M38 CLPs were similar in size and morphology to WT + DNA cargo CLPs, with only slight differences in chemical and thermal stability (Figure 2 and Figure 3). When DNA cargo was added during WT CP + M38 CLP assembly, less M38 CP was packaged in the CLPs, but was not totally excluded, suggesting that M38 CP is able to compete with DNA during CLP assembly (Figure 5). Changes in the starting concentrations of M38 CP, DNA, and WT CP and the order in which these reactants were added did affect the composition of the CLPs. Thus, M38 CP appears to be able to substitute for and compete with traditional nucleic acid cargo in this in vitro CLP assembly system.

Cargo-free assembly has been previously described in alphaviruses using the RRV 4D CP [19]. A small portion of the 4D CP is able to assemble with itself in the absence of nucleic acids. However, these 4D cargo-free CLPs are smaller (33–37 nm) than WT + DNA cargo CLPs and also display different stability properties in buffers of varying tonicity and temperature. 4D cargo-free CLPs do not disassemble at 500 mM NaCl, and the CLPs display a biphasic denaturation by DSF with fluorescence at 48 °C and 54.1 °C [19]. This type of curve suggests that there are multiple types of interactions occurring between the CPs in each CLP or that there are two populations of CLPs. Interestingly, unlike M38 CP alone, 4D CP can still assemble with DNA cargo, and actually forms WT-like assemblies in the presence of DNA cargo (data not shown). The CLPs assembled from WT CP and M38 CP have properties that are more similar to CLPs assembled from WT CP + DNA cargo and nucleocapsid cores isolated from infected cells. We anticipate the WT CP + M38 CLPs would function more like WT cores during particle budding from the plasma membrane, however that remains a question for further detailed studies.

Previous studies of alphavirus assembly have typically focused on the range of polyanionic cargoes that could be used to initiate assembly and be packaged [20,26,28,31]. In this study, we investigated the relative preference between cargoes for assembly. We found that DNA cargo was preferred over a negatively charged CP. While no sequence or structural motifs have been identified to be necessary for the in vitro assembly of alphavirus CLPs, this preference for DNA cargo suggests that factors other than electrostatic interactions are important for core assembly. The conformational changes or specific interactions between the N-terminal amino acids and the nucleic acids suggest that specific interactions are important for assembly, even outside the cell. This preference could be due to many different factors, such as the length and flexibility of the cargo or differences in interactions between CPs and cargo or CP-CP interactions. Flexibility is likely a factor during assembly because ssDNA, but not dsDNA, can be used to initiate assembly [20]. In the WT + M38 CLPs we assume the acidic N-terminus of M38 is interacting with the basic N-terminus of WT CP, while the ordered C-terminal domains of both CPs maintain interactions to form the hexameric and pentameric lattice of the core. However, it is also possible that the M38 protein is encapsidated inside the CLP particle, like the nucleic acid. We have no evidence to either support or refute these models.

The substitution of a protein substrate for DNA cargo during alphavirus CLP assembly has multiple potential applications. Many viral systems have been engineered for biotechnology applications. For example, it has been demonstrated that CCMV and P22 can be modified to act as contrast agents for magnetic resonance imaging [44,45]. P22 has been further engineered to encapsulate a variety of exogenous proteins, including other viral proteins as a vaccine strategy [46,47,48,49,50,51,52]. Alphaviruses themselves have been engineered for biotechnology purposes. Multiple alphaviruses, including Venezuelan equine encephalitis and Semliki forest virus have been engineered to treat cancer or provide protection against other viral infections with some of these treatments advancing to clinical trials [53,54]. 

Previous work has shown that alphavirus CLPs can be coated with viral glycoproteins to form infectious virus-like particles [25,30]. CLPs that were assembled from WT CP and different polyanionic cargo were always “full” and there was no evidence of extra space or the ability of multi-cargo packaging inside the CLP (data not shown). From our studies using 4D CP and M38 CP, we can begin to identify which features are mechanistically required to engineer CLPs of a specific size and shape. In this current study, we determined M38 could assemble with WT CP and form a relatively empty particle. Furthermore, this CLP could be filled with nucleic acid if all three components (M38, WT CP, and nucleic acid) were added during assembly and, the amount of cargo inside the CLP could be modulated. This work adds to our current knowledge of functional domains required for self-assembly and cargo packaging. All contributing to the long-term goal of using alphaviruses for biotechnological applications. 

## Figures and Tables

**Table 1:** Reaction conditions affect the number and density of CLPs.

Sample	Ratio	% Sucrose in Peak CLP Fraction *	Normalized Relative CLPs **
**WT + DNA Cargo**	1:1	18.68–20.00	0.59
**WT + M38**	0.5:1	14.59	0.26
	1:1	14.40	0.56
	2:1	14.63	0.73
**WT + (M38 + DNA Cargo)**	0.5:1:1	17.62	0.17
	1:1:1	18.93	0.40
	2:1:1	18.65	1.00
**(WT + DNA Cargo) + M38**	1:1:1	18.94	0.42
**(WT + M38) + DNA Cargo**	1:1:1	17.27	0.43

* Peak fractions are the fractions determined to have the most CP signal from densitometry analysis. The % sucrose for those fractions was determined by measuring the refractive index using a refractometer and converting the value to percent sucrose. ** The total number of CLPs was determined by DLS in biological triplicates. The values were averaged and then normalized throughout all samples. The values provided in both columns are an average of at least three biological replicates, except for WT + DNA Cargo sucrose percentages (2 replicates).

## Abbreviations

CP	capsid protein
M38	Minus 38
WT	wild-type
CLP	core-like particle
DLS	dynamic light scattering
TEM	transmission electron microscopy
RRV	Ross River Virus
DSF	differential scanning fluorimetry

## References

[1] Chen R., Mukhopadhyay S., Merits A., Bolling B., Nasar F., Coffey L.L., Powers A., Weaver S.C., ICTV Report Consortium (2018). Ictv virus taxonomy profile: Togaviridae. J. Gen. Virol..

[2] Cheng R.H., Kuhn R.J., Olson N.H., Rossmann M.G., Choi H.K., Smith T.J., Baker T.S. (1995). Nucleocapsid and glycoprotein organization in an enveloped virus. Cell.

[3] Jose J., Snyder J.E., Kuhn R.J. (2009). A structural and functional perspective of alphavirus replication and assembly. Future Microbiol..

[4] Zhang R., Hryc C.F., Cong Y., Liu X., Jakana J., Gorchakov R., Baker M.L., Weaver S.C., Chiu W. (2011). 4.4 a cryo-em structure of an enveloped alphavirus venezuelan equine encephalitis virus. EMBO J..

[5] Tang J., Jose J., Chipman P., Zhang W., Kuhn R.J., Baker T.S. (2011). Molecular links between the e2 envelope glycoprotein and nucleocapsid core in sindbis virus. J. Mol. Biol..

[6] Kostyuchenko V.A., Jakana J., Liu X., Haddow A.D., Aung M., Weaver S.C., Chiu W., Lok S.M. (2011). The structure of barmah forest virus as revealed by cryo-electron microscopy at a 6-angstrom resolution has detailed transmembrane protein architecture and interactions. J. Virol..

[7] Mukhopadhyay S., Zhang W., Gabler S., Chipman P.R., Strauss E.G., Strauss J.H., Baker T.S., Kuhn R.J., Rossmann M.G. (2006). Mapping the structure and function of the e1 and e2 glycoproteins in alphaviruses. Structure.

[8] Sun S., Xiang Y., Akahata W., Holdaway H., Pal P., Zhang X., Diamond M.S., Nabel G.J., Rossmann M.G. (2013). Structural analyses at pseudo atomic resolution of chikungunya virus and antibodies show mechanisms of neutralization. Elife.

[9] Mendes A., Kuhn R.J. (2018). Alphavirus nucleocapsid packaging and assembly. Viruses.

[10] Lulla V., Kim D.Y., Frolova E.I., Frolov I. (2013). The amino-terminal domain of alphavirus capsid protein is dispensable for viral particle assembly but regulates rna encapsidation through cooperative functions of its subdomains. J. Virol..

[11] Forsell K., Suomalainen M., Garoff H. (1995). Structure-function relation of the nh2-terminal domain of the semliki forest virus capsid protein. J. Virol..

[12] Forsell K., Griffiths G., Garoff H. (1996). Preformed cytoplasmic nucleocapsids are not necessary for alphavirus budding. EMBO J..

[13] Jose J., Przybyla L., Edwards T.J., Perera R., Burgner J.W., Kuhn R.J. (2012). Interactions of the cytoplasmic domain of sindbis virus e2 with nucleocapsid cores promote alphavirus budding. J. Virol..

[14] Lee S., Owen K.E., Choi H.K., Lee H., Lu G., Wengler G., Brown D.T., Rossmann M.G., Kuhn R.J. (1996). Identification of a protein binding site on the surface of the alphavirus nucleocapsid and its implication in virus assembly. Structure.

[15] Skoging U., Vihinen M., Nilsson L., Liljestrom P. (1996). Aromatic interactions define the binding of the alphavirus spike to its nucleocapsid. Structure.

[16] Wilkinson T.A., Tellinghuisen T.L., Kuhn R.J., Post C.B. (2005). Association of sindbis virus capsid protein with phospholipid membranes and the e2 glycoprotein: Implications for alphavirus assembly. Biochemistry.

[17] Hong E.M., Perera R., Kuhn R.J. (2006). Alphavirus capsid protein helix i controls a checkpoint in nucleocapsid core assembly. J. Virol..

[18] Perera R., Navaratnarajah C., Kuhn R.J. (2003). A heterologous coiled coil can substitute for helix i of the sindbis virus capsid protein. J. Virol..

[19] Rayaprolu V., Moore A., Wang J.C., Goh B.C., Perilla J., Zlotnick A., Mukhopadhyay S. (2017). Length of encapsidated cargo impacts stability and structure of in vitro assembled alphavirus core-like particles. J. Phys. Condens Matter.

[20] Tellinghuisen T.L., Hamburger A.E., Fisher B.R., Ostendorp R., Kuhn R.J. (1999). In vitro assembly of alphavirus cores by using nucleocapsid protein expressed in escherichia coli. J. Virol..

[21] Warrier R., Linger B.R., Golden B.L., Kuhn R.J. (2008). Role of sindbis virus capsid protein region ii in nucleocapsid core assembly and encapsidation of genomic rna. J. Virol..

[22] Linger B.R., Kunovska L., Kuhn R.J., Golden B.L. (2004). Sindbis virus nucleocapsid assembly: Rna folding promotes capsid protein dimerization. RNA.

[23] Tellinghuisen T.L., Perera R., Kuhn R.J. (2001). In vitro assembly of sindbis virus core-like particles from cross-linked dimers of truncated and mutant capsid proteins. J. Virol..

[24] Perera R., Owen K.E., Tellinghuisen T.L., Gorbalenya A.E., Kuhn R.J. (2001). Alphavirus nucleocapsid protein contains a putative coiled coil alpha-helix important for core assembly. J. Virol..

[25] Cheng F., Mukhopadhyay S. (2011). Generating enveloped virus-like particles with in vitro assembled cores. Virology.

[26] Cheng F., Tsvetkova I.B., Khuong Y.L., Moore A.W., Arnold R.J., Goicochea N.L., Dragnea B., Mukhopadhyay S. (2013). The packaging of different cargo into enveloped viral nanoparticles. Mol. Pharm..

[27] Wang J.C., Chen C., Rayaprolu V., Mukhopadhyay S., Zlotnick A. (2015). Self-assembly of an alphavirus core-like particle is distinguished by strong intersubunit association energy and structural defects. ACS Nano.

[28] Wengler G., Wengler G., Boege U., Wahn K. (1984). Establishment and analysis of a system which allows assembly and disassembly of alphavirus core-like particles under physiological conditions in vitro. Virology.

[29] Mukhopadhyay S., Chipman P.R., Hong E.M., Kuhn R.J., Rossmann M.G. (2002). In vitro-assembled alphavirus core-like particles maintain a structure similar to that of nucleocapsid cores in mature virus. J. Virol..

[30] Snyder J.E., Azizgolshani O., Wu B., He Y., Lee A.C., Jose J., Suter D.M., Knobler C.M., Gelbart W.M., Kuhn R.J. (2011). Rescue of infectious particles from preassembled alphavirus nucleocapsid cores. J. Virol..

[31] Goicochea N.L., De M., Rotello V.M., Mukhopadhyay S., Dragnea B. (2007). Core-like particles of an enveloped animal virus can self-assemble efficiently on artificial templates. Nano Lett..

[32] Pfeiffer P., Hirth L. (1974). Aggregation states of brome mosaic virus protein. Virology.

[33] Lavelle L., Gingery M., Phillips M., Gelbart W.M., Knobler C.M., Cadena-Nava R.D., Vega-Acosta J.R., Pinedo-Torres L.A., Ruiz-Garcia J. (2009). Phase diagram of self-assembled viral capsid protein polymorphs. J. Phys. Chem. B.

[34] Atabekov J., Nikitin N., Arkhipenko M., Chirkov S., Karpova O. (2011). Thermal transition of native tobacco mosaic virus and rna-free viral proteins into spherical nanoparticles. J. Gen. Virol..

[35] Bruckman M.A., Hern S., Jiang K., Flask C.A., Yu X., Steinmetz N.F. (2013). Tobacco mosaic virus rods and spheres as supramolecular high-relaxivity mri contrast agents. J. Mater. Chem. B.

[36] Bruckman M.A., VanMeter A., Steinmetz N.F. (2015). Nanomanufacturing of tobacco mosaic virus-based spherical biomaterials using a continuous flow method. ACS Biomater. Sci. Eng..

[37] van Eldijk M.B., Wang J.C., Minten I.J., Li C., Zlotnick A., Nolte R.J., Cornelissen J.J., van Hest J.C. (2012). Designing two self-assembly mechanisms into one viral capsid protein. J. Am. Chem. Soc..

[38] Ceres P., Zlotnick A. (2002). Weak protein-protein interactions are sufficient to drive assembly of hepatitis b virus capsids. Biochemistry.

[39] Johnson M.C., Scobie H.M., Ma Y.M., Vogt V.M. (2002). Nucleic acid-independent retrovirus assembly can be driven by dimerization. J. Virol..

[40] Schindelin J., Arganda-Carreras I., Frise E., Kaynig V., Longair M., Pietzsch T., Preibisch S., Rueden C., Saalfeld S., Schmid B. (2012). Fiji: An open-source platform for biological-image analysis. Nat. Methods.

[41] Artimo P., Jonnalagedda M., Arnold K., Baratin D., Csardi G., de Castro E., Duvaud S., Flegel V., Fortier A., Gasteiger E. (2012). Expasy: Sib bioinformatics resource portal. Nucleic Acids Res..

[42] Singh S., Zlotnick A. (2003). Observed hysteresis of virus capsid disassembly is implicit in kinetic models of assembly. J. Biol. Chem..

[43] Parent K.N., Suhanovsky M.M., Teschke C.M. (2007). Phage p22 procapsids equilibrate with free coat protein subunits. J. Mol. Biol..

[44] Liepold L., Anderson S., Willits D., Oltrogge L., Frank J.A., Douglas T., Young M. (2007). Viral capsids as mri contrast agents. Magn. Reson. Med..

[45] Usselman R.J., Qazi S., Aggarwal P., Eaton S.S., Eaton G.R., Russek S., Douglas T. (2015). Gadolinium-loaded viral capsids as magnetic resonance imaging contrast agents. Appl. Magn. Reson..

[46] Kang S., Uchida M., O’Neil A., Li R., Prevelige P.E., Douglas T. (2010). Implementation of p22 viral capsids as nanoplatforms. Biomacromolecules.

[47] Sharma J., Uchida M., Miettinen H.M., Douglas T. (2017). Modular interior loading and exterior decoration of a virus-like particle. Nanoscale.

[48] Qazi S., Miettinen H.M., Wilkinson R.A., McCoy K., Douglas T., Wiedenheft B. (2016). Programmed self-assembly of an active p22-cas9 nanocarrier system. Mol. Pharm..

[49] O’Neil A., Prevelige P.E., Basu G., Douglas T. (2012). Coconfinement of fluorescent proteins: Spatially enforced communication of gfp and mcherry encapsulated within the p22 capsid. Biomacromolecules.

[50] Patterson D.P., Prevelige P.E., Douglas T. (2012). Nanoreactors by programmed enzyme encapsulation inside the capsid of the bacteriophage p22. ACS Nano.

[51] Patterson D.P., Rynda-Apple A., Harmsen A.L., Harmsen A.G., Douglas T. (2013). Biomimetic antigenic nanoparticles elicit controlled protective immune response to influenza. ACS Nano.

[52] Schwarz B., Morabito K.M., Ruckwardt T.J., Patterson D.P., Avera J., Miettinen H.M., Graham B.S., Douglas T. (2016). Viruslike particles encapsidating respiratory syncytial virus m and m2 proteins induce robust t cell responses. ACS Biomater. Sci. Eng..

[53] Lundstrom K. (2018). Self-replicating RNA viruses for RNA therapeutics. Molecules.

[54] Lundstrom K. (2019). Rna viruses as tools in gene therapy and vaccine development. Genes.

